# Inconsistent condom use between serodifferent sexual partnerships to the human immunodeficiency virus*

**DOI:** 10.1590/1518-8345.3059.3222

**Published:** 2019-12-05

**Authors:** Renata Karina Reis, Elizabete Santos Melo, Nilo Martinez Fernandes, Marcela Antonini, Lis Aparecida de Souza Neves, Elucir Gir

**Affiliations:** 1 Universidade de São Paulo, Escola de Enfermagem de Ribeirão Preto, PAHO/WHO Colaborating Centre for Nursing Research Development, Ribeirão Preto, SP, Brazil.; 2 Scholarship holder at the Coordenação de Aperfeiçoamento de Pessoal de Nível Superior (CAPES), Brazil.; 3 Fundação Oswaldo Cruz, Instituto de Pesquisa Clínica Evandro Chagas, Rio de Janeiro, RJ, Brazil.; 4 Scholarship holder at the Programa Institucional de Bolsas de Iniciação Científica (PIBIC), Brazil.; 5 Prefeitura Municipal de Ribeirão Preto, Programa de Doenças Sexualmente Transmissíveis, Aids, Tuberculose e Hepatites Virais, Ribeirão Preto, SP, Brazil.

**Keywords:** Condoms, Unsafe Sex, HIV Infections, Disease Prevention, Nursing Care, HIV Seropositivity, Preservativos, Sexo sem Proteção, Infecções por HIV, Prevenção de Doenças, Cuidados de Enfermagem, Soropositividade para HIV, Condones, Sexo Inseguro, Infecciones por VIH, Prevención de Enfermedades, Atención de Enfermería, Seropositividad para VIH

## Abstract

**Objective:**

to analyze predictors of inconsistent condom use among HIV-positive people with sexual immunodeficiency virus serodifferent sexual partnership.

**Method:**

cross-sectional, analytical study with a consecutive non-probabilistic sample consisting of people living with the human immunodeficiency virus with serodifferent sexual partnership and who were in outpatient clinical follow-up. Data were collected through individual interviews guided by a semi-structured questionnaire and subsequently analyzed with bivariate analysis and logistic regression.

**Results:**

Seven variables were independently associated with inconsistent condom use. Schooling less than 11 years of schooling (4.9 [2.4-10.1]), having multiple partnerships (5.0 [1.3-19.6]), using alcohol (2.1 [1.1 -4.4]) or other drugs (2.8 [1.2-6.3]), do not receive advice from a healthcare professional (2.0 [1.1-3.9]), have no knowledge of treatment as prevention (3.0 [1,2-6,9]) and not knowing that undetectable viral load reduces the risk of human immunodeficiency virus transmission (3.8 [1,1-13,7]) were predictors for inconsistent condom use.

**Conclusion:**

The study showed that psychosocial factors interfere with consistent condom use between serodifferent partnerships. Thus, it is highlighted that there is a need for comprehensive interventions that include the integration of clinical and psychosocial care.

## Abstract (pt)

**Objetivo:**

analisar os preditores do uso inconsistente do preservativo entre pessoas soropositivas com parceria sexual sorodiferentes ao vírus da imunodeficiência humana.

**Método:**

estudo transversal, analítico com amostra consecutiva não probabilística que foi constituída por pessoas vivendo com o vírus da imunodeficiência humana com parceria sexual sorodiferente e que estavam em seguimento clínico ambulatorial. Os dados foram coletados por meio de entrevista individual norteadas por questionário semiestruturado e analisados com análise bivariada e regressão logística.

**Resultados:**

Identificou-se que sete variáveis foram independentemente associadas com o uso inconsistente do preservativo. Escolaridade menor que 11 anos de estudo (4,9 [2,4-10,1]), ter múltiplas parcerias (5,0 [1,3-19,6]), usar álcool (2,1 [1,1-4,4]) ou outras drogas (2,8 [1,2-6,3]), não receber aconselhamento com profissional de saúde (2,0 [1,1-3,9]), não ter conhecimento sobre tratamento como prevenção (3,0 [1,2-6,9]) e desconhecer que carga viral indetectável reduz o risco de transmissão do vírus da imunodeficiência humana (3,8 [1,1-13,7]), foram preditores para o uso inconsistente do preservativo.

**Conclusão:**

o estudo evidenciou que fatores psicossociais interferem no uso consistente do preservativo entre parcerias sorodiferentes. Assim, destaca-se que há necessidade de intervenções abrangentes que incluam a integração do cuidado clínico e psicossocial.

## Abstract (es)

**Objetivo:**

analizar a los predictores del uso inconsistente del preservativo entre personas seropositivas con asociación sexual serodiferentes al virus de la inmunodeficiencia humana.

**Método:**

estudio transversal, analítico con muestra consecutiva no probabilística que fue constituida por personas viviendo con el virus de la inmunodeficiencia humana con asociación sexual serodiferente y que estaban en seguimiento clínico de ambulatorio. Los datos fueron recogidos por medio de encuesta individual orientada por cuestionario semiestructurado y fueron analizados con análisis bivariado y regresión logística.

**Resultados:**

Se identificó que siete variables fueron independientemente asociadas con el uso inconsistente del preservativo. Escolaridad menor que 11 años de estudio (4,9 [2,4-10,1]), ter múltiples asociaciones (5,0 [1,3-19,6]), usar alcohol (2,1 [1,1-4,4]) u otras drogas (2,8 [1,2-6,3]), no recibir consejo con profesional de salud (2,0 [1,1-3,9]), no tener conocimiento sobre tratamiento como prevención (3,0 [1,2-6,9]) y desconocer que carga viral indetectable reduce el riesgo de transmisión del virus de la inmunodeficiencia humana (3,8 [1,1-13,7]), fueron predictores para el uso inconsistente del preservativo.

**Conclusión:**

el estudio evidenció que factores psicosociales interfieren en el uso consistente del preservativo entre asociaciones serodiferentes. Así, se destaca que hay necesidad de intervenciones amplias que incluyan la integración del cuidado clínico y psicosocial.

## Introduction

The scientific and technological advances regarding the health of people living with HIV (PLHIV), especially in the therapeutic field, with the advent of antiretroviral therapy (ART), caused changes in the life expectancy and perspective of these individuals^(^ 1 ^-^ 2 ^)^. 

Thus, the framing of human immunodeficiency virus (HIV) infection as a chronic disease, from access to ART, generated a new paradigm^(^ 3 ^)^ with implications related to the integral health care of these people, since initially, the concern of health services was only to contain the infection.

These changes made it possible for PLHIV to reconstruct their life projects in various aspects, especially in the affective-sexual context, with the establishment of new relationships with sexual partnerships^(^ 4 ^)^, including HIV seronegative. 

HIV serodifferent couples - when one partner is HIV-positive and the other HIV-negative^(^ 5 ^)^ - have specific vulnerabilities and are at greater risk of becoming infected with HIV^(^ 6 ^)^. 

In Brazil, despite efforts to interrupt the chain of transmission obtained through the provision of effective antiretroviral therapy, there is still a significant portion of PLHIV that has not reached undetectable viral load (VL), which is one of the factors considered most important for reducing sexual transmission of HIV^(^ 7 ^)^. 

The UNAIDS Joint United Nations Program on HIV / AIDS “cascade” of care projects, by 2030, an ambitious target for the treatment of HIV / AIDS in which 90% of people are diagnosed with HIV; 90% of these people get treated and 90% of them reach viral suppression^(^ 8 ^)^. 

Data related to the cascade of continuous HIV care in Brazil showed that by the end of 2015 there were 827,000 PLHIV in the country of which 715,000 (87%) were diagnosed; 95% of the diagnosed (677 thousand) had been linked to some health service and 83% of the linked (565 thousand) were retained in this service; 80% of those retained (455 thousand) were on ART and only 50% of those on ART (410 thousand) had suppressed VL^(^ 9 ^)^.

Thus, it is noteworthy that it is necessary to advance the fight against the AIDS epidemic in Brazil, because challenges still persist for the approach of sexuality and sexual behavior in the continuous care of PLHIV. 

Historically, efforts to prevent infection have focused on reducing the risk of HIV transmission among seronegative individuals or those with unknown serological status and minimizing the needs and important role of PLHIV^(^ 10 ^)^ as if the discovery of infection by itself would ensure changes in the preventive practices and behaviors and vulnerability they faced. 

Measures and strategies to prevent sexual transmission of HIV have changed over the course of the epidemic mainly due to scientific advances in the world. The findings defined new forms of prevention that were referred to in Brazil as “combined prevention”^(^ 10 ^)^.

It is the association of behavioral, biomedical and structural intervention strategies^(^ 10 ^)^ focusing on reducing the transmission of HIV infection in various sectors, as it is known that a single prevention strategy is insufficient to control multiple HIV epidemics in the world and in Brazil^(^ 6 ^)^.

It is understood that, in the context of combined prevention, strategies aim to reduce the adherence gaps present in the use of classic preventive methods in isolation, such as the use of only male or female condoms as a preventive method.

To this end, the Ministry of Health has drafted the Combined Prevention Mandala, which outlines all possible strategies that may be effective for preventing HIV infection^(^ 11 ^)^. Thus, during consultations with Specialized Care Services (SCS), users are advised that no single method of prevention can reduce HIV infection^(^ 11 ^)^.

Condoms are considered an important component of the combined approach to HIV prevention and, when used consistently and correctly, are highly effective at preventing sexual transmission of the virus and other sexually transmitted infections (STIs)^(^ 11 ^)^. 

In Brazil, condoms are freely distributed by the Unified Health System (UHS) and are a safe, low-cost barrier method with no adverse effects. It should be stimulated among PLHIV in combination with other methods such as treatment as prevention (TPT), pre-exposure prophylaxis (PrEP) and post-exposure prophylaxis (PEP)^(^ 11 ^)^, as inconsistent use increases the risk of HIV reinfection, just as exposure to STI increases the infectivity of the virus and therefore its transmissibility^(^ 12 ^)^.

In this sense, inconsistent male condom use has been described as prevalent among PLHIV with different types of sexual partnerships - both regular and casual, with negative HIV status or unknown HIV status, in different regions of the world^(^ 13 ^-^ 19 ^)^. 

However, according to the World Health Organization (WHO), most studies on serodifferent couples have been conducted in sub-Saharan Africa, and knowledge gaps exist in other regions of the world ^(^ 5 ^)^, resulting in insufficient emphasis on providing counseling for couples and supporting testing of HIV-positive partners. 

In Brazil, some studies ^(^ 20 ^-^ 21 ^)^ addressed the relationship of PLHIV in a serodifferent partnership and the use of the male condom, however, they were limited only to evaluate the use and not the factors associated with it. 

Therefore, the aim of this study was to analyze the predictors of inconsistent male condom use among HIV-positive people with HIV-negative sexual partnership.

## Method

This is a cross-sectional and analytical study that was conducted in the five Specialized Care Services (SCS) in a city in the interior of São Paulo. 

Participated in the PVHIV study of both sexes, regardless of the stage of HIV infection, which met the inclusion criteria: being aware of the diagnosis of HIV infection; being over 18 years old; being in outpatient clinical follow-up at the services; have an active sex life and sexual partnership regardless of HIV status in the last six months. Those in confinement situations such as persons deprived of liberty and institutionalized, as well as residents of nursing homes, were excluded. 

The sampling plan adopted was by simple random sampling in which prevalence parameters were selected by estimating that 62% of people living with HIV have an active sex life after diagnosis, as described in another study conducted in the municipality ^(^ 22 ^)^, relative error of 10% and significance level of 5% and a very large total population (N = 10,000, for example), which indicated the need for a sample size of 235. However, to reach this number, 397 PLHIV were interviewed, 286 of whom had HIV serodifferent partnerships. 

Data was collected from July 2016 to July 2017 through individual interviews, in specialized outpatient rooms, using a questionnaire built and validated specifically for this data collection. Participants were invited before or after the medical appointment, and the interviews lasted a minimum of 20 and a maximum of 30 minutes and were conducted by five duly trained research assistants.

After a wide search in the literature, it was considered that, in Brazil, until the moment of collection, there were no scales that evaluated the knowledge about the preventive strategies, the beliefs about the infectivity and the sexual transmission of HIV, as well as the preventive behavior of PLHIV. Then, a specific instrument based on national and international literature was elaborated to respond to the objectives of this study, which was submitted to validation as to form and content by three nurses and a psychologist, all specialists in the study theme. 

Experts in the area of HIV infection were invited via email to the committee of experts. The process took place by analyzing the instrument as to the general impression, objective, content, relevance, verbal language and inclusion of new questions. After the return, the necessary adjustments were made to achieve the research objectives.

The instrument consisted of sociodemographic variables: age (in years); gender (male, female); education (in complete years of study); profession / occupation; employment relationship (yes or no); color (white, brown, black, indigenous) and clinical: time of diagnosis of HIV infection (in years), plus presence of infectious markers: symptoms of STI (discharge, warts, blisters and genital ulcers) according to Brazilian protocol ^(^ 6 ^)^ (yes or no); viral load in the last six months (detectable or undetectable), with a value below 40 copies / mL considered undetectable. 

Regarding the behavioral, practical and knowledge aspects of HIV transmission and counseling about sexual prevention of HIV by the health team, the following behavioral variables were adopted: type of sexual partnership (casual, fixed or casual and fixed); sexual partnership number; alcohol use before sexual intercourse (yes or no); alcohol use during sexual intercourse (yes or no); period of couple formation (before or after the knowledge of HIV infection); HIV serological diagnosis of sexual partnership (seropositive, seronegative or unknown). Belief in sexual transmission of HIV was assessed by the following question: Do you believe that if your viral load is low, you decrease the risk of HIV transmission? (yes / no / don’t know), complemented by the questions: did you reveal the diagnosis for the current sexual partnership (yes or no)?; talk to your partner about condom use (yes or no) and male condom use (always, sometimes and never)? 

The variables selected to assess the knowledge and advice received by the health care team on HIV sexual prevention were: did you receive guidance on risk of sexual transmission of HIV (yes / no) ?; Looking for information on preventing sexual transmission of HIV? (Yes No)?; Did you receive counseling from your healthcare team about sexual transmission of HIV (yes / no)? Knowledge about the effectiveness of preventive treatment has been assessed by the question: antiretroviral (ARV) treatment, reducing the amount of circulating virus in the body (viral load) and making it undetectable, is effective in preventing HIV transmission to the partner? (yes / no / don’t know). 

Inconsistent condom use was defined as “yes” for those participants who reported that they never or sometimes used condoms (versus “no”, ie “always used condoms”) for the past six months.

Data was statistically analyzed using IBM® Statistical Package for Social Sciences (SPSS) software, version 23, and R (R Core Team software, version 3.4.1) software. 

Descriptive statistics were performed to characterize the participants and analytical to verify the association between the study variables through the chi-square test. To assess the influence of independent variables on inconsistent condom use (yes / no), logistic regression analysis was used. The category “yes” was adopted as a reference in all cases. For the analysis, a significance level of 5% (α = 0.05) was adopted.

The study was submitted and appreciated by the Ribeirão Preto Municipal Health Department, which was favorable to its accomplishment. Subsequently, it was submitted to and approved by the Research Ethics Committee of the Ribeirão Preto College of Nursing, following the recommendations of Resolution 466/12 of the National Health Council, under Opinion No. 2,369,369. 

## Results

The study included 286 PLHIV who had a serodifferent sexual partnership, with an average age of 41.2 years, ranging from 18 to 73 years. Table 1 shows that the sample consisted predominantly of men (68.6%) and of these 37.8% were men who have sex with men (MSM). It was found that the majority (51.4%) of respondents had elementary education and about 67.1% were in the formal or informal job market.

**Table 1:** Distribution of people living with HIV* in serodifferent partnership, according to sociodemographic and clinical variables. Ribeirão Preto, SP, Brazil, 2016-2017

Variables		n	%
Sexual orientation			
	Heterosexual woman	90	31.5
	Heterosexual man	88	30.8
	MSM†	108	37.8
Age group (years)			
	18 – 24	23	8.0
	25 – 34	59	20.6
	35 – 44	100	35.0
	≥ 45	104	36.4
Education (complete years of study)			
	< 11	147	51.4
	≥ 11	139	48.6
Color			
	White	151	52.8
	Black	132	46.2
	Others		
Work situation			
	Emloyed	192	67.1
	Unemployed	59	20.6
	Others	33	11.5
	Do not work (on leave)	02	0.7
Family income (minimum wages‡)			
	Up to 1	59	20.6
	2- 3	133	46.5
	≥ 3	94	32.9
HIV diagnosis time* (years)			
	< 2 – 4.9	105	36.7
	≥ 5	181	63.3
Infectiousness markers			
STI symptoms§			
	Yes	40	14.0
	No	246	86.0
Viral load			
	Detectable	70	24.5
	Indetectable	216	75.5

* HIV = Human Immunodeficiency Virus;

† MSM = men who have sex with men;

‡ minimum wages = minimum wage equivalent to R $ 937.00, in the period from 2016 to 2017, in Brazil;

§ STI = Sexually Transmitted Infections

It was also identified that 58% of PLHIV revealed the diagnosis of seropositivity for sexual partnership. Relationships, mostly (74.1%), were established after the discovery of HIV infection, and approximately half (50.3%) of participants reported not talking to their partner about condoms. 

Among the participants, 72.7% claimed to have had risky behaviors and practices in the last six months. Of this total, it was found that 44.1% had sexual intercourse under the influence of alcohol, 34.4% had sexual intercourse with multiple partners and 29.0% reported having sex without a condom or making inconsistent use of it, as shown in the table 2.

**Table 2:** Distribution of behaviors and practices of people living with HIV* in serodifferent partnership. Ribeirão Preto, SP, Brazil, 2016-2017

Variables		N	%
Alcohol use before sexual intercourse			
	Yes	126	44.1
	No	160	55.9
Drug use before sexual intercourse			
	Yes	59	20.9
	No	227	79.4
Partner uses alcohol before sexual intercourse			
	Yes	143	50.0
	No	143	50.0
Partner uses drugs before sexual intercourse			
	Yes	56	19.5
	No	227	79.4
Number of Partners			
	One	179	62.6
	Multiple	107	34.4
Revealed Diagnosis for Sex Partnership			
	Yes	166	58.0
	No	120	42.0
Talk to partner about condom use			
	Yes	142	49.7
	No	144	50.3
Inconsistent condom use			
	Yes	83	29.0
	No	203	71.0
Met Partner Before HIV Discovered *			
	Yes	74	25.9
	No	212	74.1

* HIV = Human Immunodeficiency Virus

Table 3 describes the bivariate analysis of sociodemographic and clinical factors associated with inconsistent male condom use among PLWHA who have a serodifferent sexual partnership. Schooling was found to be associated with inconsistent condom use (p = <0.001). 

**Table 3:** Sociodemographic and clinical factors associated with inconsistent condom use with HIV-negative sexual partnership* among people living with HIV†. Ribeirão Preto, SP, Brazil, 2016 - 2017

Variables		Condom use with HIV-negative sexual partnership*			
		Consistent 203 (71 %)	Inconsistent 83 (29.0%)	Total 286 (100)	p-value
Sexual orientation					
	Heterosexual woman	58 (28.6)	32 (38.6)	90 (31.5)	0.070
	Heterosexual man	60 (29.6)	28 (33.7)	88 (30.8)	
	MSM‡	85 (41.9)	23 (27.7)	108 (37.8)	
Age group (years)					
	18 – 24	16 (7.9)	07 (8.4)	23 (8.0)	0.987
	25 – 34	42 (20.7)	17 (20.5)	59 (20.6)	
	35 – 44	70 (34.5)	30 (36.1)	100 (35.0)	
	≥ 45	75 (36.9)	29 (34.9)	104 (36.4)	
Education (years)					
	< 11	84 (41.4)	63 (75.9)	147 (51.4)	<0.001
	≥ 11	119 (58.6)	20 (24.1)	139 (46.9)	
Skin color					
	White	102 (50.2)	49 (61.3)	151 (53.4)	0.147
	Black	20 (9.9)	09 (11.2)	29 (10.2)	
	Others	81 (39.9)	22 (27.5)	103 (36.4)	
HIV diagnosis time† (years)					
	< 2-4.9	71 (35.0)	34 (41.0)	105 (36.7)	0.340
	≥ 5	132 (65.0)	49 (59.0)	181 (63.3)	
Infectiousness markers					
Viral load					
	Detectable	47 (23.2)	23 (27.7)	70 (24.5)	0.416
	Indetectable	156 (76.8)	60 (72.3)	216 (75.5)	
STI symptoms§					
	Yes	25 (12.3)	15 (18.1)	246 (86.0)	0.203
	No	178 (87.7)	68 (81.9)	40 (14.0)	

* HIV-negative = Human immunodeficiency virus negative serology;

† HIV = Human Immunodeficiency Virus;

‡ MSM = men who have sex with men;

§ STI = Sexually Transmitted Infections

Regarding behavioral variables, it was found that the number of partners (p = 0.048), alcohol use (p = <0.001) and other drugs (p = <0.001) during sexual intercourse were associated with inconsistent condom use. 

Table 4 also presents another bivariate analysis of the following behavioral variables: knowledge of undetectable viral load in HIV transmission (p = <0.001); the search for information on prevention methods on the internet (p = 0.026); not searching for information on prevention methods (p = <0.001); receiving counseling at the health facility (p = 0.023) and talking to a partner about condom use (p = <0.001) also showed a significant association with inconsistent condom use in HIV-positive women who have an HIV-negative partnership.

**Table 4:** Behavioral, knowledge and counseling factors associated with inconsistent condom use with HIV-negative sexual partnership* of people living with HIV†. Ribeirão Preto, SP, Brazil, 2016 - 2017

Variables		Condom use with HIV-negative sexual partnership*			
		Consistent 203 (71.0)	Inconsistent 83 (29.0)	Total 286 (100)	p-value
Partnership Type					
	Main	112 (55.2)	37 (44.6)	149 (52.1)	0.225
	Casual	82 (40.4)	40 (48.2)	122 (42.7)	
	Multiple Partnerships	09 (4.4)	06 (7.2)	15 (5.2)	
Number of Partners					
	01	136 (67.0)	43 (51.8)	179 (62.6)	0.048
	02 – 5	42 (20.7)	27 (32.5)	69 (24.1)	
	≥ 5	25 (12.3)	13 (15.7)	38 (13.3)	
Alcohol use during sex					
	Yes	73 (36.0)	53 (63.9)	126 (44.1)	<0.001
	No	130 (64.0)	30 (36.1)	160 (55.9)	
Drug use during sex					
	Yes	25 (12.3)	34 (41.0)	59 (20.6)	<0.001
	No	178 (87.7)	49 (59.0)	227 (79.4)	
Belief about HIV transmission†					
	Yes	101 (49.8)	52 (60.2)	153 (53.5)	<0.001
	No	98 (48.3)	18 (21.7)	116 (40.6)	
	Don’t know	04 (20.0)	13 (15.7)	17 (5.9)	
Search information about prevention methods on the internet					
	Yes	90 (44.3)	25 (30.1)	115 (40.2)	0.026
	No	113 (55.7)	58 (69.9)	171 (58.2)	
Does not seek information on prevention methods					
	Yes	19 (9.4)	29 (34.9)	48 (16.8)	<0.001
	No	184 (90.6)	54 (65.1)	238 (83.2)	
Received advice from healthcare professional					
	Yes	29 (14.3)	13 (15.7)	42 (14.3)	0.023
	No	174 (85.7)	67 (80.7)		
Revealed diagnosis					
	Yes	122 (60.1)	44 (53.0)	166 (58.0)	0.270
	No	81 (39.9)	39 (47.0)	120 (42.0)	
Talk to partner about condom use					
	Yes	116 (57.1)	26 (31.3)	142 (49.7)	<0.001
	No	87 (42.9)	57 (68.7)	144 (50.3)	
Knowledge about treatment as prevention					
	Yes	163 (80.3)	50 (60.2)	213 (74.5)	0.001
	No	26 (12.8)	17 (20.5)	43 (15.0)	
	Don’t know	14 (6.9)	16 (19.3)	30 (10.5)	

* HIV-negative = Human immunodeficiency virus negative serology;

† HIV = Human Immunodeficiency Virus

In multivariate analysis, seven variables were independently associated with inconsistent condom use. Individuals who reported less than 11 years of schooling (4.9 [2.4-10.1]), have a steady and casual partner (5.0 [1.3-19.6]), use alcohol (2 , 1 [1.0-4.4]) or other drugs (2.8 [1.2-6.3]), do not receive counseling from a healthcare professional (2.0 [1.0-3.9]), have no knowledge of treatment as prevention (3.0 [1.2-6.9]) and who do not know if the burden Undetectable viral infection reduces the risk of HIV transmission (3.8 [1.0-13.7]) were predictors for inconsistent condom use, as shown in Table 5.

**Table 5:** Predictors associated with inconsistent condom use among people living with HIV* in serodifferent partnerships. Ribeirão Preto, SP, Brazil, 2016 - 2017

Variables		aOR† [95% CI‡]	p-value
Education (years)			
	< 11	4.9 [2.4-10.1]	<0.001
	≥ 11	Ref.§	
Type of partnership			
	Main	Ref.§	
	Casual	0.7 [0.3-1.4]	0.384
	Main and casual	5.0 [1.3-19.6]	0.019
Alcohol use during sex			
	Yes	Ref.§	0.034
	No	2.1 [1.1-4.4]	
Drug use during sex			
	Yes	Ref.§	0.012
	No	2.8 [1.2-6.3]	
Received advice from healthcare professional			
	Yes	Ref.§	0.030
	No	2.0 [1.1-3.9]	
Knowledge about treatment as prevention			
	Yes	Ref.§	
	No	3.0 [1.2-6.9]	0.010
	Don’t know	1.4 [0.5-3.7]	0.453
Knowledge about HIV transmission*			
	Yes	Ref.§	
	No	0.40 [0.20-0.82]	0.012
	Don’t know	3.8 [1.1-13.7]	0.040

* HIV = Human Immunodeficiency Virus;

† aOR = Adjusted Odds ratio;

‡ CI = Confidence Interval;

§ Ref. = Reference values

## Discussion

In Brazil, there is a lack of official epidemiological and behavioral data on HIV serodifferent couples, in varied contexts of the relationship, either with fixed and / or casual partnerships. This fact highlights its invisibility in services, health policies, as well as between social movements and researchers^(^ 23 ^-^ 24 ^)^. 

In this study, it was found that few people (29%) reported inconsistent condom use with negative or unknown HIV status, which may be corroborated by other investigations that found rates of 28.7% respectively^(^ 22 ^)^ and 20.7%^(^ 25 ^)^ of inconsistent condom use among PLHIV. 

In fact, although the prevalence of risky sexual behavior decreases after the discovery of HIV infection^(^ 17 ^)^, Studies conducted among PLHIV reported inconsistent condom use with HIV-negative or unknown sex partners in different regions of the world^(^ 15 ^-^ 18 ^,^ 26 ^-^ 28 ^)^.

In this sense, it is noteworthy that, in clinical practice, in addition to all the assistance already provided, one should also pay attention to the investigation of behaviors and practices beyond the inconsistent use of condoms, as this is fundamental for the identification of individuals in risk of non-adherence or suboptimal adherence to effective preventive strategies. Other studies have also investigated this issue that may contribute to the risk of HIV transmission to HIV-negative sexual partnership and also reinfection by HIV or acquisition of another STI by HIV-positive partners^(^ 27 ^-^ 30 ^)^.

The results indicate that having a lower educational level was a predictor for inconsistent condom use in the individuals who were part of the study. These results resemble those of other studies, which revealed that a higher level of education is associated with regular condom use^(^ 31 ^-^ 32 ^)^. In addition, another study showed that as the level of education increases, the chance of engaging in risky sexual practices decreases^(^ 4 ^)^.

Low education conditions access to information and the ability to assimilate and understand the guidance received in health services and directly interfere with health behavior and the possibility of adopting protective practices for themselves and others^(^ 33 ^)^.

Better access to information, understanding and awareness about preventing sexual transmission of HIV can foster sexual negotiation and increased condom adherence. 

In this study, it was observed that having a casual partner was associated with inconsistent condom use (p = <0.001). And PLHIV who have both fixed and casual partnerships simultaneously are 5.0 times more likely (CI = 95%, 1.3-19.6, p = 0.019) to engage in unprotected sex compared to those who have only single and fixed partnerships. This may be related to the fact that those who have had multiple sexual partners may not disclose their status to their partners, which was also described in a study in Ethiopia^(^ 34 ^)^. Thus, the type of bond and the affective-sexual relationship interfere with condom adherence. 

Nursing consultations during the clinical follow-up of PLHIV should include a comprehensive sexual history approach that includes aspects related to sexual partnerships for the promotion of educational interventions and counseling. Health professionals should identify aspects of the relationship and the types of sexual partnerships. Sexual history assessment, which includes the type and number of sexual partnerships, should be a vital part of any health education and counseling session. 

A multicenter study of both heterosexual and homosexual serodifferent couples identified 11 cases of HIV transmission among people who had casual partnerships outside of a fixed partnership relationship^(^ 35 ^)^.

Thus, observing HIV transmission outside the relationship with fixed partnerships increases the importance of counseling nurses assessing types of partnerships and inconsistent condom use^(^ 13 ^)^. 

Including information about sexual partnerships in risk management and counseling will help to better understand individual risk profiles and to plan appropriate intervention strategies for HIV prevention.

Another predictor found in this study was that using alcohol during sexual intercourse is 2.1 more likely to have unprotected sex with an HIV-serodifferent sexual partnership. This result is in line with several other studies in the literature that have shown the association between alcohol use and risky sexual behavior among PLHIV^(^ 36 ^-^ 37 ^)^. Among serodifferent partnerships, this behavior is particularly worrying, mainly because it exposes seronegative partners to HIV exposure. 

Alcohol has been associated with disinhibition in which people may be more likely to engage in riskier sexual behaviors ^(^ 38 ^)^. In fact, alcohol use is considered a risk factor for HIV infection because of its interference with adherence to prevention methods such as condoms, impaired ability or willingness to be assertive when negotiating condom use with a resistant partner and decreased perceptions of possible negative consequences of condomless sex^(^ 38 ^)^.

Systematic review investigating the effects of alcohol consumption on unprotected sexual intentions showed that participants who consumed alcohol were less likely to perform communication and sexual negotiation skills^(^ 39 ^)^.

In addition, alcohol and other drug use is also associated with poor adherence to ART, which may lead to a higher risk of therapeutic failure and, consequently, to sustained non-suppression of viral load^(^ 40 ^-^ 42 ^)^. 

Therefore, interventions directed at the association between alcohol and sexual behavior should highlight the negative influence of alcohol on decision making for protected sex, particularly with occasional partners^(^ 43 ^)^. 

For this, health services should offer prevention programs that address coprevalent conditions^(^ 44 ^)^, counseling is needed for people using psychoactive substances, including risk reduction interventions and management of sexual transmission of infection. 

Another predictor associated with inconsistent condom use was that health professionals did not receive guidance on preventing sexual transmission of HIV. This result strengthens the importance of health professionals and particularly nurses in reducing inequalities in HIV literacy. Moreover, this result leads to a reflection on the relevance of increasing investments in studies of educational interventions, which allow the establishment of causal relationships between health literacy, prevention literacy and preventive behaviors, so that more effective strategies can be established that are in line with the needs of PLHIV. 

The nurse plays an important role in facilitating the communication processes associated with the promotion of health literacy by assessing comprehension, individual motivating factors, barriers to understanding as well as in clear communication with individuals, making health information readable and by adapting the message to their cultural and linguistic needs and promoting more appropriate health decision-making^(^ 45 ^)^.

In this sense, nurses and health staff should implement and evaluate basic health literacy promotion strategies to improve knowledge and skills for adherence to an effective HIV prevention strategy. 

The results of this study showed that limited knowledge of PLHIV living in the context of sero-discordance about “treatment as prevention” and misinformation that undetectable viral load does not reduce the risk of HIV transmission were predictors of unprotected sex. 

A study in the UK among HIV-affected communities also found that most participants expressed little understanding and confidence in the effect of undetectable viremia on the risk of HIV transmission^(^ 46 ^)^.

In Brazil, a study conducted in the Federal District, which described the perceptions of PLHIV in the context of serodiscordance, on the prevention of HIV transmission, identified difficulties in implementing the most recent advances and scientific discoveries in practice. The authors pointed out that, despite the broadening of the range of existing preventive strategies in the context of combined prevention, PLHIV had insufficient knowledge to use new preventive inputs^(^ 47 ^)^, highlighting the importance of disseminating this information in people’s daily lives.

Thus, one of the biggest challenges facing these couples relates to knowledge about advances related to HIV prevention, adoption and negotiation of these preventive strategies. Many people living with HIV or even those who are most vulnerable are unaware of the benefits of “treatment as prevention” as a prevention strategy, the concept of combined prevention and risk management of sexual transmission of HIV. 

Insufficient knowledge of PLHIV about the use of new prevention tools, including “treatment as prevention”, aimed at suppressing CV (undetectable = not transferable) and the use of PrEP or PEP by the HIV-negative partner to compromise the adoption of effective preventive practices by exposing their sexual partnerships to HIV. 

In this regard, PLHIV and their sexual partnerships should receive counseling and education with serodifferent couples about these HIV prevention options in isolation or their association with other risk management and prevention methods such as PrEP and condoms^(^ 48 ^)^. 

Condoms, when used consistently and correctly, are highly effective at preventing sexual transmission of HIV and other STIs and are considered an important component of a comprehensive approach to HIV prevention^(^ 13 ^)^. Reducing condom use increases the risk of exposure to STIs and HIV infectivity and, therefore, their transmissibility^(^ 14 ^)^. 

In the context of combined prevention, the male condom remains a method of protection against STI and HIV infection, and it remains critical to expand its access to the entire population as a priority action^(^ 11 ^)^, especially for people at risk of exposure to HIV and those who are in HIV serodifferent partnership^(^ 6 ^)^. 

To this end, it is necessary to overcome the exclusively rational logic of prevention work, which is reflected in a purely prescriptive attitude of condom use, also called “latex fundamentalism”, in which it is imposed without the necessary dialogue and reflection for overcoming the difficulties of its use^(^ 21 ^)^. 

More innovative approaches that dialogue with the autonomy of PLHIV and their sexual partnerships are needed. 

Therefore, considering that prevention options for HIV-serodifferent couples are expanding and becoming more widely available, educational interventions can be a potentially useful tool to help them explore options, make decisions, and identify prevention methods that are best suited to fit their reality and life context^(^ 49 ^)^ and can help PLHIV not only recognize the importance of the male condom, but also to be informed about the existence of other methods that can be combined with the same, enabling them to manage their own risks^(^ 11 ^)^.

Finally, it is noteworthy that this study had some limitations, such as the non-random sample and the fact that information on sexual behavior and condom use was obtained through the patient’s report, which may lead to a bias of answer, since it seeks to report what is considered “more correct” than it is actually practiced, which may lead to an underestimation of the risk. 

## Conclusion

This study showed that 29% of PLHIV, in a serodifferent partnership, reported inconsistent condom use. Individual factors - having less education, type of partnership and affective-sexual relationship (multiplicity of sexual partnership); psychosocial - use psychoactive substances, such as alcohol or other drugs, and related to health services - not receiving advice from a healthcare professional, being unaware of treatment as prevention, and unaware that undetectable viral load reduces the risk of HIV transmission were associated with inconsistent condom use.

These results highlight the challenge for HIV prevention and reveal the need for a broader approach to PLHIV care, which includes assessing their sexual partners, desires, fears and difficulties. It is essential that there is a change in current care practice, centered on the biological model, which emphasizes only aspects related to medication adherence and prescriptive condom use. 

The male condom, while effective for HIV prevention, is insufficient if used alone to overcome the challenges posed by the epidemic, as important barriers are still observed among PLHIV who have HIV-serodifferent sexual partners and are outpatient clinical follow-up in specialized care services. 

Comprehensive health care for these individuals implies the need for comprehensive interventions that include the integration of clinical and psychosocial care, addressing factors that favor non-adherence to preventive strategies and risky behavior, such as alcohol and other drug use. 

It is recommended that the health team recognize the urgent need to address the discussion regarding the adoption of alternative proposals for condom use within the context of combined prevention, implementing couple counseling, testing, and support for drug disclosure. HIV diagnosis to sexual partnership, so that the vulnerability of PLHIV seronegative sexual partnerships can be reduced.
